# Real-Time Motion Tracking for Indoor Moving Sphere Objects with a LiDAR Sensor

**DOI:** 10.3390/s17091932

**Published:** 2017-08-23

**Authors:** Lvwen Huang, Siyuan Chen, Jianfeng Zhang, Bang Cheng, Mingqing Liu

**Affiliations:** 1 College of Information Engineering, Northwest A&F University, Xianyang 712100, China; sy.chen@hotmail.com (S.C.); 18392105294@163.com (M.L.); 2 Key Laboratory of Agricultural Internet of Things, Ministry of Agriculture, Xianyang 712100, China; 3 College of Mechatronic Engineering and Automation, National University of Defense Technology, Changsha 410073, China; chengbang32@163.com

**Keywords:** 3D LiDAR, object tracking, Kalman filter, adaptive particle filter

## Abstract

Object tracking is a crucial research subfield in computer vision and it has wide applications in navigation, robotics and military applications and so on. In this paper, the real-time visualization of 3D point clouds data based on the VLP-16 3D Light Detection and Ranging (LiDAR) sensor is achieved, and on the basis of preprocessing, fast ground segmentation, Euclidean clustering segmentation for outliers, View Feature Histogram (VFH) feature extraction, establishing object models and searching matching a moving spherical target, the Kalman filter and adaptive particle filter are used to estimate in real-time the position of a moving spherical target. The experimental results show that the Kalman filter has the advantages of high efficiency while adaptive particle filter has the advantages of high robustness and high precision when tested and validated on three kinds of scenes under the condition of target partial occlusion and interference, different moving speed and different trajectories. The research can be applied in the natural environment of fruit identification and tracking, robot navigation and control and other fields.

## 1. Introduction

### 1.1. Application of LiDAR

Light Detection and Ranging (LiDAR) technology provides realistic 3-dimensional (3D) image information and has been widely utilized in various fields [1]. LiDAR sensors are commonly used in perception for autonomous vehicles because of their high accuracy, speed, and range. These characteristics make the sensors suitable for integration into the perception layer of controllers which have the capacity to avoid collisions with unpredicted obstacles [2]. LiDAR technology is also applied to field Autonomous Land Vehicles (ALVs) to detect potential obstacles. With a novel 3D LiDAR setup, the blind area around the vehicle is greatly reduced and the density of LiDAR data is greatly improved, which are critical for ALVs [3]. In addition to autonomous land vehicle applications, LiDAR is also used for navigation of unmanned aircraft systems [4]. The authors combined LiDAR to automatically identify ground objects that pose navigation restrictions such as airports and high-rises.

Meanwhile, in the field of agriculture and forestry, by combining field and LiDAR data in forests with coexisting evergreen and deciduous species, researchers modelled common forest stand variables (height, diameter, volume and biomass) with high accuracy [5]. At the same time, LiDAR point clouds data is used for comparative classification analysis of post-harvest growth detection in precision agriculture [6], while other researchers have proposed a new approach for discriminating maize and weed plants from soil surface, evaluating the accuracy and performance of a LiDAR sensor for vegetation detection using distance and reflection values [7].

In other respects, LiDAR technology contributes to detect flood protection structures, natural or artificial in river floodplains and in coastal zones [8]. Also, 3D information derived from image dense matching or airborne LiDAR is very effective for building change detection [9]. Furthermore, for many robotics and intelligent vehicle applications, detection and tracking multiple objects based on LiDAR is one of the most important components [10].

### 1.2. Tracking Algorithms Based on LiDAR

LiDAR systems are commonly used for pedestrian recognition in ALVs, compared with cameras and can provide accurate range information and larger field of view [11]. For years, Kalman filters (KF) and Monte Carlo particle filters (PF) have been the two commonly used approaches to estimate motions of a target. In some early works, Song et al. [12] proposed a novel sparse learning-based object tracking algorithm utilizing 3D LiDAR data to realize moving object tracking of vehicles. The 3D point clouds acquired from LiDAR are first resampled on a virtual image plane, where the hypothesis of the targets is generated under the particle filtering framework. Guo et al. [13] proposed a pedestrian tracking algorithm initializing a KF to predict the possible position of the pedestrian centroid in the future frame. Meanwhile, Dewan et al. [14] have presented a novel model-free approach for detecting and tracking dynamic objects in 3D LiDAR scans obtained by a moving sensor. They sequentially detected multiple motions in the scene and segment objects using a Bayesian approach. Allodi et al. [15] have presented an obstacle detection, tracking and fusion algorithm which allows to reconstruct the environment surrounding the vehicle. An Unscented Kalman Filter (UKF) managing a variable number of observations, arbitrarily composable, allows to correctly address the combined tracking and fusion challenge. Moreover, Wasik et al. [16] have proposed a method based on the detection of circular features with least-squares fitting and filtering out outliers using a map-based selection. They have improved the estimate of the relative robot position and reduce its uncertainty by feeding measurements into a KF, resulting in an accurate tracking system.

For detecting and tracking moving objects in more complex cases, an occupancy grid tracking system based on particles [17] has been proposed. The proposed occupancy grid tracking solution can be classified as using the Descartes probability model of the reverse sensor and it generates a fully dynamic grid. To resolve ambiguities in complex dynamic scenes, Tuncer et al. [18] proposed a novel method for integrated tracking and segmentation of 3D LiDAR data with a non-parametric Bayesian method to combine segmentation and tracking components. In [19], Asvadi et al. proposed a 3D object tracking algorithm using a 3D-LiDAR, an RGB (Red, Green, Blue) camera and INS (Inertial Navigation System) (GPS (Global Position System)/IMU (Inertial Measurement Unit)) sensors data by analyzing sequential 2D-RGB, 3D point-cloud, and the ego-vehicle’s localization data and outputs the trajectory of the tracked object, an estimation of its current velocity, and its predicted location in the 3D world coordinate system in the next time-step while in [20], feature matching, Iterative Closest Point (ICP), Kalman filtering, and dynamic mapping are combined together to estimate motions.

As mentioned above, the KF, the PF UKF and non-parametric Bayesian are used in detecting and tracking moving targets and there is no straightforward extension of their approach to a moving spherical object.

### 1.3. Application of Kalman Filter

The KF has achieved notable success in the areas of guidance, navigation, and control of vehicles, particularly aircraft and spacecraft. Srilekha et al. [21] introduced a new technique for detecting, tracking and counting the vehicles based on Kalman filtering. Huang et al. [22] proposed the Robust Strong Tracking Cubature Kalman Filter (RSTCKF) for spacecraft attitude estimation with a quaternion constraint. Furthermore, the KF is a widely applied concept in time series analysis used in fields such as signal processing and econometrics. Jain et al. [23] investigated the use of KF to estimate and track both the laser PN (Phase Noises) and the NLPN (Nonlinear Phase Noises) in 100-Gb/s single channel coherent optical phase-modulated systems. The KF is one of the main topics in the field of robotic motion planning and control. Gulalkari et al. [24] proposed an object tracking and following six-legged robot (6LR) system that uses a Kinect camera based on KF and back-stepping control method. Lim et al. [25] proposed incorporating dead-reckoning using only encoder measurements, and a Kalman filter-based Gaussian Process to compensate the uncertainty. As for other aspects, Moon [26] developed a human skeleton tracking system using the Kalman filter framework, in which multiple Kinect sensors are used to correct inaccurate tracking data from a single Kinect sensor.

**Figure 1:**
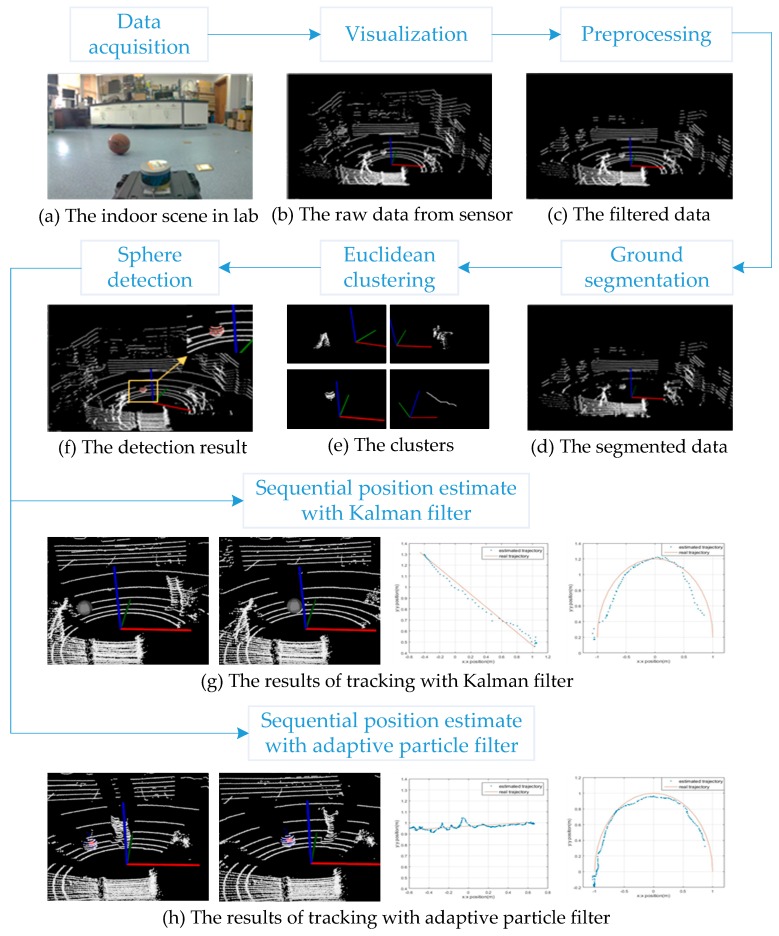
The tracking flowchart for a moving object.

In order to solve the real-time tracking process for a moving sphere at indoor environments and in the future for spherical fruit identification and positioning with varying illumination, this paper first introduces the processes of sphere detection with 3D LiDAR, and then discusses the principles of the KF and PF algorithms. Next, with experimental modeling, data analysis of two tracking methods is compared, and finally we reach a conclusion. The tracking flowchart is as shown in Figure 1 below.

## 2. Detection of Moving Spherical Object

### 2.1. The Velodyne System

**Figure 2:**
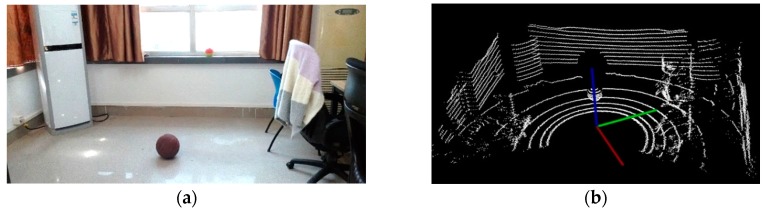
The results of visualization: (**a**) Experimental scene; (**b**) Point clouds visualization.

The Velodyne VLP-16 3D LiDAR sensor obtains a 360-degree scene capture through the rotation of its internal motor. It is composed of 16 laser beams, which scan thousands of times per second. Each beam has a fixed pitch angle. The experimental scene and its visualization result are shown in Figure 2.

### 2.2. Outliers and Noise Filtering

To reduce the calculation of segmentation after the 3D data acquisition, it is necessary to eliminate some of the noise, outliers, holes, etc. by filtering according to some motion cues. Here we remove the coordinate origin (0, 0, 0) and then use outlier filter proposed by Rusu et al. [27] which works well for indoor scenes. Firstly, we compute the average distance of each point between its nearest k neighbors. Next, we compute the mean μ, and standard deviation σ of all these distances to determine a distance threshold. The standard deviation coefficient α depends on the size of the analyzed neighborhood k. The distance threshold t will be equal to:

(1) t=μ+α×σ

**Figure 3:**
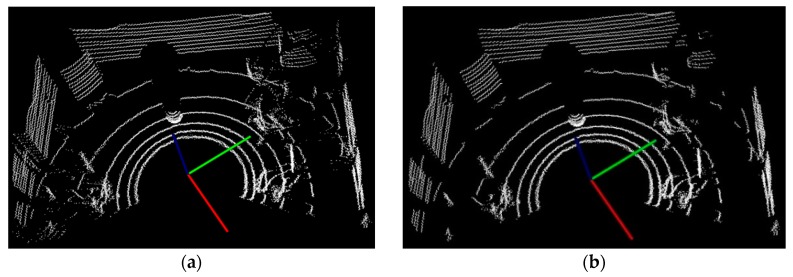
The results of outliers and noise filtering: (**a**) Before filtering; (**b**) After filtering.

In Equation (1), α is set to 1 and k is set to 30 here with the empiric and experimental threshold, especially for the indoor scene. Finally, the points can be classified as inliers or outliers if their average neighbor distance is below or above this threshold respectively. The results of filtering are shown below in Figure 3, where most of the noises and outliers marked by red ellipse are removed.

### 2.3. Fast Ground Segmentation

For the indoor object motion tracking, the ground segmentation is essential to cancelling the ground background information. The fast ground segmentation algorithm proposed by Himmelsbach et al. [28] is used to remove the ground noise and to reduce the amount of subsequent calculations, which requires less runtime and obtains good segmentation results.

#### 2.3.1. 3D Point-Cloud Data Set Mapping

**Figure 4:**
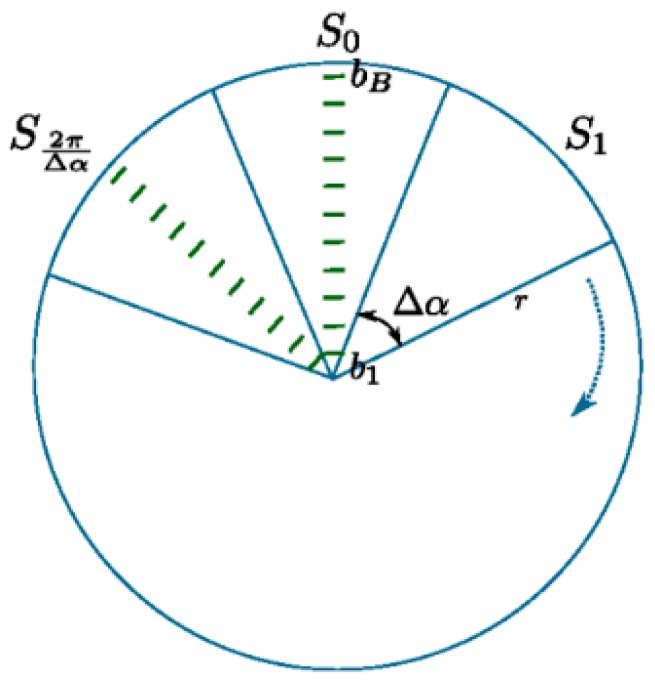
Partitioning the 3D space into segments of equal size.

1. Define the unordered 3D point clouds from a scan time t of the LiDAR sensor as $P_{t}=\left\{p_{1},\;\ldots \;\ldots ,\;p_{N_{t}}\right\}$, where $N_{t}$ denotes the number of 3D point clouds. The $p_{i}={\left(x_{i},y_{i},z_{i}\right)}^{T}$ denotes a 3D point, given by the Euclidean coordinates to the ego-coordinate system with original point at the center of the LiDAR sensor.
2. The *x*-o-*y* plane denotes a circle with a radius of R, and then cut the circle equally into multiple discrete sectors, as shown in Figure 4. The Δα denotes the angle of each sector plane, so the number of sectors M=2π/Δα.
3. $S_{i}$ represents each sector, where 1≤i≤M, so that each point can be classified into a sector plane according to its projection on the *x*-o-*y* plane, expressed as a segment ($p_{i}$):
  (2) $$\mathrm{segment}\left(p_{i}\right)=\frac{atan2\left(y_{i},x_{i}\right)}{\Delta \alpha },$$
  where $\mathrm{atan}2(y_{i},x_{i})$ represents the angle within [0, 2π) between the positive direction of *x*-axis and *x*-o-*y* plane, $y_{i}$ representing *y*-value of $p_{i}$, $x_{i}$ representing *x*-value of $p_{i}$, and Δα representing the angle of each sector plane.

We denote the set of all points mapped to the same segment s by $P_{s}$:

(3) $$P_{s}=\left\{p_{i}\in P|segment\left(p_{i}\right)=s\right\},$$

Define a mapping of all points $P_{s}$ of the same segment to one of many bins $b_{j}^{s}$, j=1…B discretizing the range component of the points, while the superscript s denotes the sector that the bin belongs to. The minimum or maximum range that a bin covers is expressed respectively by $r_{j}^{min}$ and $r_{j}^{max}$. Obviously, a point $p_{i}\in P_{s}$ maps to bin $b_{j}^{s}$:

(4) $$r_{j}^{min}\leq \sqrt{x_{i}^{2}+y_{i}^{2}}\leq r_{j}^{max},$$

The $P_{b_{j}^{s}}$ is denoted by the set of all points mapping to $b_{j}^{s}$. Given a set of $P_{b_{j}^{s}}$ of 3D points mapped to the same bin, a new set of 2D points $P_{b_{j}^{s}}^{\prime }$ is simply defined as:

(5) $$P_{b_{j}^{s}}^{\prime }=\left\{p_{i}^{\prime }|p_{i}\in P_{b_{j}^{s}}\right\},$$

where $p_{i}$ representing any point in 3D space, $p_{i}={\left(x_{i},y_{i},z_{i}\right)}^{T}$, $P_{i}^{\prime }={\left(\sqrt{x_{i}^{2}+y_{i}^{2}},z_{i}\right)}^{T}$.

All points have been mapped to a segment and a corresponding segment bin. With the above mapping method of 3D point clouds data set, sorting from small to large by distance, the processed 3D space point set is partially of order. A prototype point $p_{b_{j}^{s}}^{\prime }$ is calculated for every non-empty bin points $P_{b_{j}^{s}}^{\prime }$ whose point with lowest *z*-coordinate and most likely belonging to the ground plane.

#### 2.3.2. Fast Ground Segmentation

**Figure 5:**
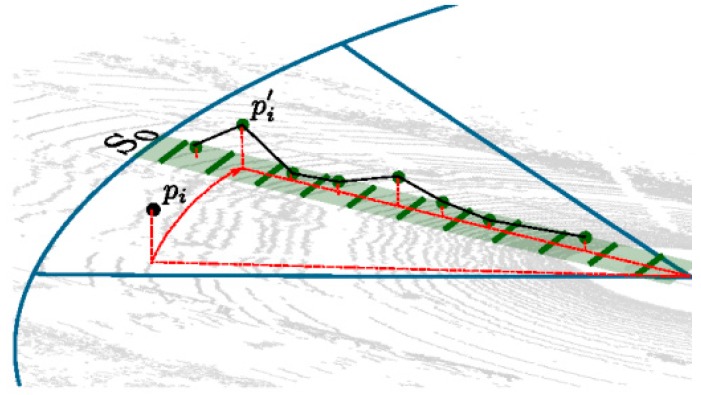
Mapping of 3D point *p* to a bin of the corresponding segment and resulting mapped point $p_{i}^{\prime }$.

On the basis of the above data mapping method of 3D point clouds data set, the ground model in a sectorial area *S* can be expressed as a set $L_{s}$ of a line segment shown in Figure 5. 

**Figure 6:**
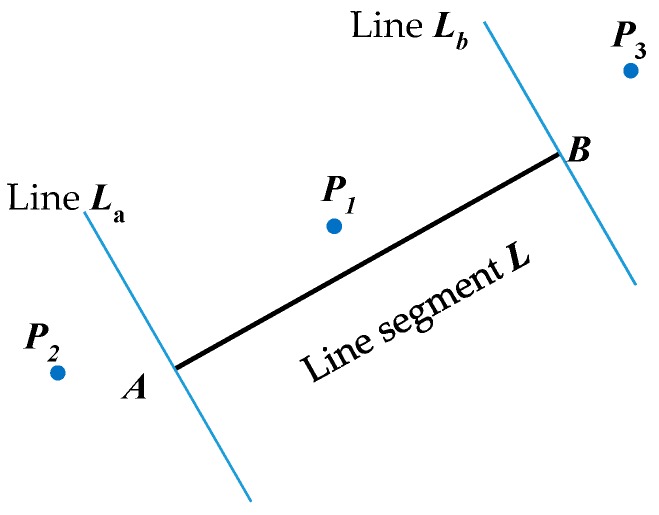
The distance between points and line segment ***L***.

Calculate the distance between a point and the line L as shown in Figure 6. Calculate the two straight lines $L_{a}$, and $L_{b}$, which pass through both ends of line segment AB, and perpendicular to line segment L. Then determine whether the point P is between the straight lines $L_{a}$, and $L_{b}$ (such as $P_{1}$ point) or outside the two lines (such as $P_{2}$ point, $P_{3}$ point). If the point is at the position $P_{1}$, the distance L and $P_{1}$ can be directly calculated. If the point is at the position $P_{2}$, then the distance between $P_{2}$ and point A is calculated. In the same way, if at point $P_{3}$, the distance between $P_{3}$ and L is equal to the distance between $P_{3}$ and point B. The process of extracting lines for a segment is expressed in Algorithm 1 as follows.

The thresholds mentioned in the algorithm are tested in the experiment with the settings shown in Table 1, while the threshold $T_{ground}$ determines whether the point belongs to a ground point. It remains to formulate necessary conditions for a line y=mx+b to be considered part of the ground plane:

1. The line’s slope m must not exceed a certain threshold $T_{m}$.
2. The line’s absolute *y*-intercept *b* must not exceed a certain threshold $T_{b}$.
3. The root mean square error of the fit must not exceed a certain threshold $T_{RMSE}$.
4. The distance of the first point of a line to the line previously fitted must not exceed $T_{dprev}$, enforcing smooth transitions between pairs of successive lines.

**Figure 7:**
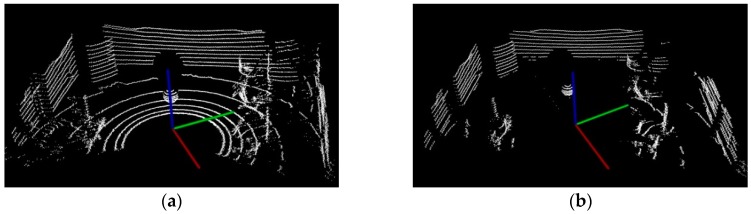
Results of fast ground segmentation: (**a**) Before segmentation; (**b**) After segmentation.

With the above bins and segments calculation, and the experimental thresholds trial selection, the fast ground segmentation results are shown in Figure 7, where the most of ground information can be effectively cancelled.
**Algorithm 1.** Extraction of lines for one segment S_S_1: $L_{s}=\emptyset$, ***c*** = 0, $p_{l}=\emptyset$ **%**
$L_{s}$ denotes a set of line set in segment S, c denotes count of loop,$\;p_{l}$ denotes a point of line2: **for *j*** = 0 **in** MAX_B **do**  % for each bin3:  **if**
$p_{b_{j}^{s}}^{\prime }\neq \emptyset$
**then**     % if there is a point mapping in $b_{j}^{s}$4:   **if** |$p_{l}$| >= 2 **then**   % if the number of points in $L_{s}$ bigger than two5:    ($m_{c},b_{c}$) = fitline($p_{l}\cup p_{b_{j}^{s}}^{\prime }$)  % line fit to get $m_{c},b_{c}$ of **L**6:    **if**
$\left|m_{c}\right|$ <= $T_{m}$ ∧ ($m_{c}$ > |$T_{m_{small}}$| ∨ $\left|b_{c}\right|$ <= $T_{b}$) ∧ fitError($m_{c},b_{c},p_{l}\cup p_{b_{j}^{s}}^{\prime }$) < $T_{\mathrm{RMSE}}$
**then**     % if match condition of thresholds7:       $p_{l}\leftarrow p_{l}\cup p_{b_{j}^{s}}^{\prime }$      % add $p_{b_{j}^{s}}^{\prime }$ to $p_{l}$8:    **else**9:       ($m_{c},b_{c}$) ← fitline ($p_{l}$)    % line fit to get $m_{c},b_{c}$ of **L**10:      $L_{s}\leftarrow L_{s}\cup \left\{\left(m_{c},b_{c}\right)\right\}$11:      $p_{l}\leftarrow \emptyset$           % clear $p_{l}$12:      ***c*** ← ***c*** + 1        % next line segment13:      ***j*** ← ***j*** − 1         % next distance14:   **else**               % if the number of points in $L_{s}$ smaller than two15:    **if**
***c*** = 0 ∨ ($p_{l}\neq \emptyset$) ∨ $distpointline\left(p_{b_{j}^{s}}^{\prime },m_{c-1},b_{c-1}\right)\leq T_{dprev}$
**then**     % if the first point or the distance of point and line match thresholds16:      $p_{l}=p_{l}\cup^{\text{​}}p_{b_{j}^{s}}^{\prime }$        % add $p_{b_{j}^{s}}^{\prime }$ to $p_{l}$

### 2.4. Object Segmentation

On the basis of fast ground segmentation to cancel the noises, the Euclidean clustering segmentation algorithm is used to segment the sphere target, and its point-cloud data needs to be trained to recognize the target.

#### 2.4.1. Euclidean Clustering of Point Clouds

**Figure 8:**
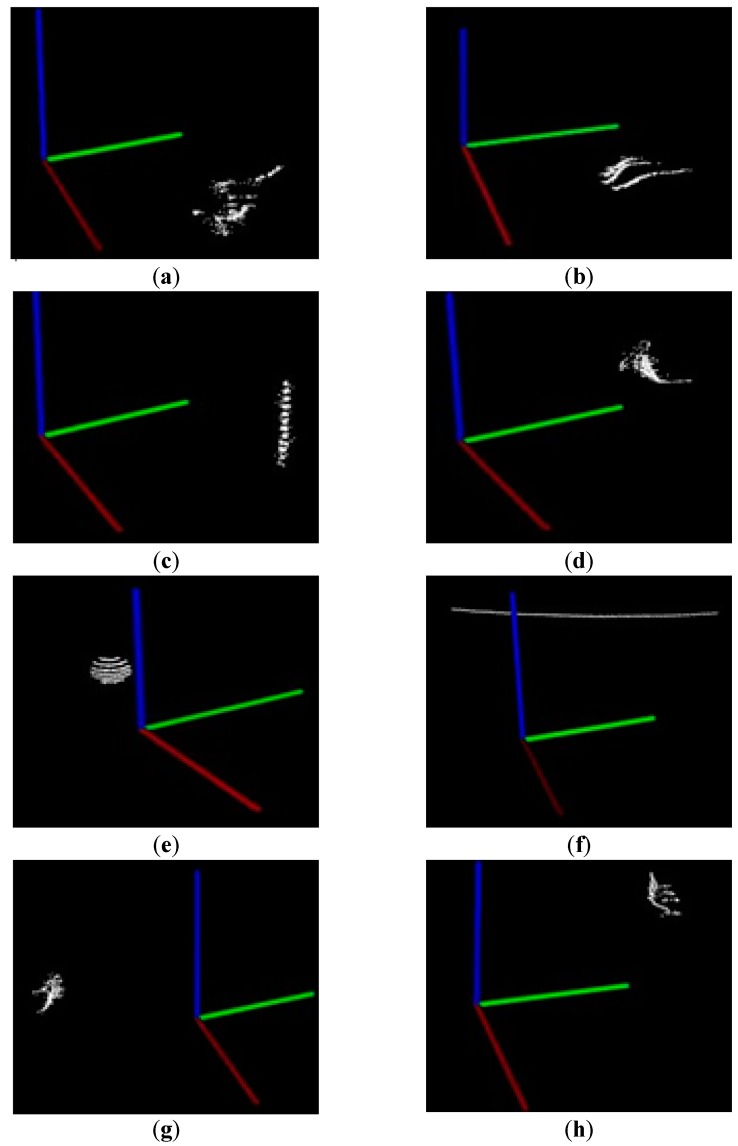
The segmentation results of Euclidean clustering: (**a**) The right chair; (**b**) The legs of the middle chair; (**c**) The pneumatic pole of the black chair; (**d**) The back of the middle chair; (**e**) The target sphere segmented; (**f**) The edge of window; (**g**) The part of chair near the air conditioning; (**h**) The back of blue chair.

The Euclidean clustering algorithm is used to segment other disperse irrelevant background sorting point clouds data of same similarity within certain threshold. The basic idea is dividing *n* points into *m* classes randomly at first, and then making the points in each class have comparatively high similarity while the points in different classes have comparatively low similarity. Then we calculate the Euclidean distance between clusters. The two clusters with a minimum distance could be merged into one cluster. We repeat the calculation of distances between clusters, and subsequent merging. With the repeated iteration until the distance between any two clusters is over than the given threshold, or the number of clusters is less than the given number, the segmentation is completed and the target sphere object is obtained. The distance threshold is set as 0.06 according to the number of sphere object’s point clouds, the minimum number of points in each class as 100, and the maximum number as 2000. The results of Euclidean clustering are shown in Figure 8, where the surrounding of the target sphere can be clearly clustered. Figure 8e shows the basic contour of the target sphere. With the Euclidean clustering, we could extract and match the moving sphere during the indoor motion tracking process.

#### 2.4.2. VFH Descriptor Extraction

**Figure 9:**
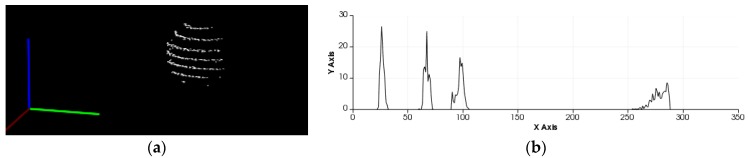
Results of VFH descriptor extraction: (**a**) Basketball’s point clouds visualization; (**b**) VFH of the basketball.

The numbers of clusters have been segmented in the process of Euclidean clustering. Subsequently the feature extraction descriptor for each cluster is employed to match the target sphere with Fast Library with Approximate Nearest Neighbors (FLANN). The feature description here describes the geometry and topology of the local or global of point clouds data sets, which can be easily understood as a point-cloud feature. The feature description of point-clouds generally consists of local and global feature descriptions. The local features describe the local geometry and shape characteristics of the point-cloud data, while the global features describe the global topological structure of the point clouds. Only for the motion tracking at the whole indoor environment to distinguish different poses, the global feature descriptor of Viewpoint Feature Histograms (VFH) is employed to estimate the feature of clusters and to extract the target. The visualization of sphere object and its VFH are shown in Figure 9.

#### 2.4.3. Feature Match of FLANN

The point clouds feature model library is constructed with the FLANN and the extraction steps are listed as follows:

(1) Acquire the point clouds data sets using the LiDAR sensor at different distance between the sphere object and sensor, and then extract VFH features for each point clouds model.
(2) Load the above VFH features into memories and convert the data into matrix format.
(3) Create the *k*-*d* (*k*-dimensional) tree with the converted matrix data, and save the index of *k*-*d* tree for the direct search match.
(4) Input the VFH feature and the index of *k*-*d*, and search the nearest neighbor along the *k*-*d* tree for the input data.
(5) Achieve the target point clouds if the difference between the searching results and VFH is less than the given threshold.

## 3. Tracking

### 3.1. Kalman Filtering

**Figure 10:**
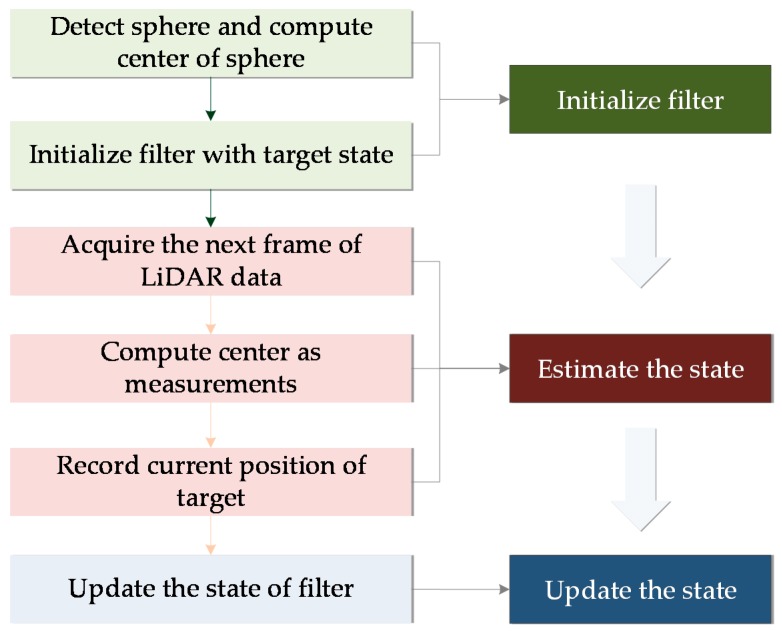
Sphere motion tracking process with KF based on LiDAR.

For the motion tracking of a moving sphere, the KF [29] provides a highly computable method in the recursive way to estimate the state of the process and minimize the estimated mean square error. The state and measurement equations are used to describe a dynamic system. The state vector $s_{k}$ of the system of time moment k is determined by both the state $s_{k-1}$ at time moment k−1 and the observed noise. The measurement vector $m_{k}$ is also determined by these two, which is by the observation function of state vector $s_{k}$ at time moment *k* and the noise. The target motion tracking process with KF is shown in Figure 10. The state variables are the positions and velocities of the sphere object in the *X*, *Y*, and *Z* coordinate, expressed as the matrix ${\left[x,y,z,v_{x},v_{y},v_{z}\right]}^{T}$ and the observed variable *z* is the objects’ position data real-time sensed by LiDAR sensor and real-time processed, denoted as ${\left[x,y,z\right]}^{T}$. Here, the state function is expressed as Equation (6):

(6) $$s_{k}=As_{k-1}+w_{k-1},$$

and the measurement function is given by Equation (7):

(7) $$m_{k}=Hm_{k}+v_{k}$$

The random variables $w_{k-1}$ and $v_{k}$ represent the process and the measurement noise respectively, denoting the noises and disturbances of the moving sensing data. They are assumed to be independent of each other, and with normal probability distributions:

(8) p(w)~N(0,Q)

(9) p(v)~N(0,R)

The matrix Q denotes the process noise covariance and R the measurement noise covariance. Here assumed that Q and R are redefined as constant shown as follows:

(10) $$Q=\left[\begin{array}{cccccc} 0.05 & 0 & 0 & 0 & 0 & 0\\ 0 & 0.05 & 0 & 0 & 0 & 0\\ 0 & 0 & 0.05 & 0 & 0 & 0\\ 0 & 0 & 0 & 0.05 & 0 & 0\\ 0 & 0 & 0 & 0 & 0.05 & 0\\ 0 & 0 & 0 & 0 & 0 & 0.05 \end{array}\right]$$

(11) $$R=\left[\begin{array}{ccc} 0.05 & 0 & 0\\ 0 & 0.05 & 0\\ 0 & 0 & 0.05 \end{array}\right]$$

Define the state variable $s_{k}$ as a six-dimensional vector shown in Equation (12):

(12) $$s_{k}={\left[x_{k},y_{k},z_{k},v_{x,k},v_{y,k},v_{z,k}\right]}^{T},$$

where $x_{k},\;y_{k},\;z_{k}$ are respectively the coordinate value of the center sphere object in the *x*, *y*, *z* coordinate system, and $v_{x,k},\;v_{y,k},\;v_{z,k}$ is respectively the speed of the center coordinates in the *x*, *y*, *z* direction.

The time interval dt between the two frames of LiDAR is only one second, which is relatively short, and the motion could be considered as a uniform motion, so the state transition matrix *A* is expressed as:

(13) $$A=\left[\begin{array}{cccccc} 1 & 0 & 0 & dt & 0 & 0\\ 0 & 1 & 0 & 0 & dt & 0\\ 0 & 0 & 1 & 0 & 0 & dt\\ 0 & 0 & 0 & 1 & 0 & 0\\ 0 & 0 & 0 & 0 & 1 & 0\\ 0 & 0 & 0 & 0 & 0 & 1 \end{array}\right]$$

The measurement vector $m_{k}$ is used to observe the center position of the moving spherical objects shown in Equation (14):

(14) $$m_{k}={\left[x_{k},y_{k},z_{k}\right]}^{T},$$

and the corresponding observation matrix is as follows:

(15) $$H=\left[\begin{array}{cccccc} 1 & 0 & 0 & -dt & 0 & 0\\ 0 & 1 & 0 & 0 & -dt & 0\\ 0 & 0 & 1 & 0 & 0 & -dt \end{array}\right].$$

The variable ${\hat{s}}_{k}^{-}$ (− represents priori, and ^ represents the estimate) denotes *k*-th priori state estimation when the *k*-th preceding state is known, and ${\hat{s}}_{k}$ is the known posterior state estimation of measurement variable $z_{k}$ at the *k*-th period.

The following Equation (16) predicts the current state value with the result of the previous finest state:

(16) $${\hat{s}}_{k}^{-}=\;A{\hat{s}}_{k-1}+w,$$

where ${\hat{s}}_{k}^{-}$ is the a priori state estimate at step *k* given knowledge of the process prior to step *k*. *A* is the state transition matrix of the system shown in Equation (13), ${\hat{s}}_{k-1}\;$is the optimal estimate at time moment *t* − 1, and *w* is the system noise following Gaussian distribution.

The uncertainty of each moment is represented by the covariance matrix *P*, and the update formula is expressed in Equation (17):

(17) $$P_{k}^{-}=AP_{t-1}A^{T}+Q,$$

where $P_{k}^{-}$ is the covariance of ${\hat{x}}_{k}^{-}$, $P_{t-1}$ is the covariance of ${\hat{x}}_{k-1}$, and *Q* is the covariance of the random signals $w_{k-1}$ and $v_{k}$. The prediction Equations (16) and (17) update time *t*.

We define a priori and a posteriori estimate error as Equations (18) and (19), respectively;

(18) $$e_{k}^{-}\equiv x_{k}-{\hat{x}}_{k}^{-},$$

(19) $$e_{k}\equiv x_{k}-{\hat{x}}_{k},$$

Then, the a priori estimated error covariance is given by Equation (20):

(20) $$P_{k}^{-}=E\left[e_{k}^{-}e_{k}^{-}{}^{T}\right],$$

and the s posteriori estimate error covariance is:

(21) $$P_{k}=E\left[e_{k}e_{k}{}^{T}\right],$$

The initial state $P_{0}$ of covariance matrix $P_{k}$ is redefined as:

(22) $$P_{0}=\left[\begin{array}{cccccc} 10 & 0.1 & 0.1 & 0.1 & 0.1 & 0.1\\ 0.1 & 10 & 0.1 & 0.1 & 0.1 & 0.1\\ 0.1 & 0.1 & 10 & 0.1 & 0.1 & 0.1\\ 0.1 & 0.1 & 0.1 & 10 & 0.1 & 0.1\\ 0.1 & 0.1 & 0.1 & 0.1 & 10 & 0.1\\ 0.1 & 0.1 & 0.1 & 0.1 & 0.1 & 10 \end{array}\right]$$

The posteriori state estimate ${\hat{s}}_{k}$ as a linear combination of an a priori estimate ${\hat{s}}_{k}^{-}$ and a weighted difference between an actual measurement $m_{k}$ and a measurement prediction $H{\hat{s}}_{k}^{-},$ calculated as shown in Equation (23):

(23) $${\hat{s}}_{k}={\hat{s}}_{k}^{-}+K_{k}\left(m_{k}-H{\hat{s}}_{k}^{-}\right),$$

where the matrix $K_{k}$ is the gain that minimizes the posteriori error covariance. The difference $\left(m_{k}-H{\hat{s}}_{k}^{-}\right)$ is the measurement innovation (residual) that reflects the discrepancy between the predicted measurement $H{\hat{s}}_{k}^{-}$ and the actual measurement $z_{k}$.

To accomplish minimization, the Equations (18) and (23) are firstly substituted into Equation (19), with a unit matrix I, then we get Equation (24):

(24) $$e_{k}=\left[I-K_{k}H\right]e_{k}^{-}-K_{k}v_{k},$$

Substituting the above Equation (24) into Equation (21), then we get:

(25) $$P_{k}=\left(I-K_{k}H\right)P_{k}^{-}{\left(I-K_{k}H\right)}^{T}+K_{k}RK_{k}{}^{T},$$

Obtaining the indicated expectations, we take the derivative of the tracking result with respect to $K_{k}$. Set the result to zero, and then solve for $K_{k}$ as below:

(26) $$K_{k}=P_{k}^{-}H^{T}{\left(HP_{k}^{-}H^{T}+R\right)}^{-1}=\frac{P_{k}^{-}H^{T}}{HP_{k}^{-}H^{T}+R},$$

With the equations above, the smaller the observed noise covariance *R*, the larger the gain $K_{k}$. The smaller the covariance $P_{k}^{-}$, the smaller the gain $K_{k}$. The Kalman gain $K_{k}$ works on two aspects, firstly, it weighs the size of the a priori estimated error of the covariance$\;P_{k}^{-}\;$and the observed noise covariance matrix *R* to determine the more convincing model between the prediction and observation model; secondly, it transforms the representation form of the residuals from the observation domain to the state domain.

To make the KF rung down till the end of the whole system running process, the covariance ${\hat{x}}_{k}$ at state t needs to be updated as follows:

(27) $$P_{k}=\left(I-K_{k}H\right)P_{k}^{-},$$

After the time updating calculation of the Equations (14) and (19) and the measurement updating Equations (20), (23) and (24), the whole Kalman tracking process repeats again. A posteriori estimation of Equation (20) obtained from the previous calculation of Equation (18) is taken as the a priori estimation of Equation (24) of the next computation. The whole process of KF is shown in Algorithm 2 as follows.
**Algorithm 2.** Kalman Filter**Input:**$\;m_{k}$,  % object position for time step t from sensor**Output:**
${\hat{s}}_{k}$,  % a position estimation of object1: **initialize**
*t*, ${\hat{s}}_{k-1}$, *A*, *P*, *Q*, *R*  % *t* represents prediction time moment, ${\hat{s}}_{k-1}$ is the known posterior       % state estimation at time moment *k* − 1; A represents state transition matrix;       % *P* represents the covariance matrix, *Q* denotes covariance of the random        % signals, and ***R*** is the matrix of observation noise covariance.2: **if** filterStop = false **then**  % end with convergence of click the ‘stop’ button.3:  ${\hat{s}}_{k}^{-}\leftarrow A{\hat{s}}_{k-1}$    % calculate predicting position estimation, according to Equation (16)4:  P ← APA^T^ + Q  % calculate priori covariance matrix, according to Equation (17)5:  K ← PC^T^(CPC^T^ + R)  % calculate Kalman Gain matrix, according to Equation (26)6:  ${\hat{s}}_{k}$ ← ${\hat{s}}_{k}^{-}$ + K($m_{k}$ − C${\hat{s}}_{k}^{-}$)  % calculate optimal estimation value, according to Equation (23)7:  P ← (I − KC)P  % calculate ${\hat{x}}_{k}$
covariance, according to Equation (27), ***I*** denotes unit matrix8:  ***t*** ← ***t*** + 19: **end if**

### 3.2. Particle Filtering

Particle filtering is a non-parametric Monte Carlo method used to simulate the realization of the recursive Bayesian filter, which is applicable to any state space model for the non-linear non-Gaussian case, and its accuracy can reach the optimal estimate. Filtered particles are possibilities to describe the target state. The purpose of filtering is the most probable state of the filtered target. In the Bayesian estimation theory, the current state of the target is estimated using the previous state and the current measured value. The arbitrary probability distribution $p(x_{k})$ can be Monte Carlo approximated using the discrete particle set as follows:

(28) $$p\left(x_{k}|y_{1:k}\right)\approx \Sigma_{i=1}^{N_{k}}w_{k}^{\left(i\right)}\delta \left(x_{k}-x_{k}^{\left(i\right)}\right),$$

where $x_{k}^{\left(i\right)}$, $w_{k}^{\left(i\right)}$, $N_{k}$ are respectively expressed as particle state, weight and total number under k time, where δ is Dirac’s delta function.

The most basic and common PF implementation framework is Sequential Importance Sampling and Resampling (SISR) or Sampling Importance Resampling (SIR) filter, and the algorithm is shown below:

Step 1: For *i* = 1, 2, …, *N*, Initializing the particle set, k=0:

Generating the sampled particles ${\left\{x_{0}^{\left(i\right)}\right\}}_{i=1}^{N}$ based on the priori distribution $p\left(x_{0}\right);$

Step 2: For k=1, 2, …, N, Executing the follow steps circularly:

Sequential importance sampling: for i=1,2,…, N, generating the sampled particles ${\left\{{\tilde{x}}_{k}^{\left(i\right)}\right\}}_{i=1}^{N}$ from the importance probability density, then calculating the particle weights, finally normalizing the weights so that the sum of the weights of the particles is 1;

Resampling: resampling the particles set $\left\{{\tilde{x}}_{k}^{\left(i\right)},{\tilde{w}}_{k}^{\left(i\right)}\right\}$, and the resampled set is $\left\{x_{k}^{\left(i\right)},1/N\right\}$;

Printing: calculating the estimated state value: ${\hat{x}}_{k}=\Sigma_{i=1}^{N}{\tilde{x}}_{k}^{\left(i\right)},{\tilde{w}}_{k}^{\left(i\right)}$.

Sequential importance sampling is the basis of particle filtering, which applies the sequential analysis method in statistics to the Monte Carlo method, so as to realize the recursive estimation of the probability density of posterior filtering. Assumed that the importance probability density function $q(x_{0:k}|y_{1:k})$ can be decomposed into:

(29) $$q\left(x_{0:k}|y_{1:k}\right)=q(x_{0:k-1}|y_{1:k-1})q(x_{k}|x_{0:k-1},y_{1:k}),$$

Let the system state be a Markov process, and the given system state is independent of each observation so that there is:

(30) $$p\left(x_{0:k}\right)=p\left(x_{0}\right)\prod_{i=1}^{k}p(x_{i}|x_{i-1}),$$

(31) $$p\left(y_{1:k}|x_{1:k}\right)=\prod_{i=1}^{k}p(y_{i}|x_{i}),$$

The recursive form of the posterior probability density function can be expressed as:

(32) $$\begin{array}{cc} p\left(x_{0:k}|Y_{k}\right) & =\frac{p(y_{k}|x_{0:k},Y_{k-1})p(x_{0:k}|Y_{k-1})}{p(y_{k}|Y_{k-1})}\\ & =\frac{p(y_{k}|x_{0:k},Y_{k-1})p(x_{k}|x_{0:k-1},Y_{k-1})p(x_{0:k}|Y_{k-1})}{p(y_{k}|Y_{k-1})}\\ & =\frac{p(y_{k}|x_{k})p(x_{k}|x_{k-1})p(x_{0:k-1}|Y_{k-1})}{p(y_{k}|Y_{k-1})} \end{array}$$

In the update phase, the particles’ weights are recalculated according to the likelihood function $p\left(x_{0:k}|Y_{k}\right)$:

(33) $$\begin{array}{cc} w_{k}^{\left(i\right)} & \infty \frac{p(x_{k}^{\left(i\right)}|Y_{k})}{q(x_{k}^{\left(i\right)}|Y_{k})},\\ & =\frac{p(y_{k}|x_{k}^{\left(i\right)})p(x_{k}^{\left(i\right)}|x_{k-1}^{\left(i\right)})p(x_{0:k-1}^{\left(i\right)}|Y_{k-1})}{q(x_{k}^{\left(i\right)}|x_{0:k-1}^{\left(i\right)},Y_{k})q(x_{0:k-1}^{\left(i\right)}|Y_{k-1})}\\ & =w_{k-1}^{\left(i\right)}\frac{p(y_{k}|x_{k}^{\left(i\right)})p(x_{k}^{\left(i\right)}|x_{k-1}^{\left(i\right)})}{q(x_{k}^{\left(i\right)}|x_{0:k-1}^{\left(i\right)},Y_{k})} \end{array}$$

In general, it is necessary to normalize the weight of the particle:

(34) $${\tilde{w}}_{k}^{\left(i\right)}=\frac{w_{k}^{\left(i\right)}}{\Sigma_{i=1}^{N}w_{k}^{\left(i\right)}},$$

This results in an approximate representation of the posterior probability density function expressed by Equation (32). In practical application, the use of too many samples will result in a sharp increase in the computational complexity and the deterioration of the performance of the particle filter. However, it is very difficult to correctly approximate the posterior probability with a small amount of sampling, and the resampling process may also lead to particle deficiency. Therefore, it is necessary to determine the appropriate sampling quantity and improve the efficiency of sampling according to the state of the system, under the condition of ensuring the diversity of the particles. In this paper, adaptive particle filter based on Kullback-Leibler Distance (KLD) sampling proposed by Fox [30] is adopted to resample the particles.

The core idea of the KLD sampling is that in each iteration of the particle filter, using the probability 1−δ to make the error between the true posterior probability and the estimated probability density based on the sample less than *ε*:

(35) $$n=\frac{1}{2\epsilon }X_{k-1,1-\delta }^{2}$$

where δ is set as 0.99 and ϵ as 0.2. So that the number of resampled samples is determined. The error is determined by calculating the KLD. The KLD is used to represent the approximation error between the two probability distributions p and q:

(36) $$K\left(p,q\right)=\Sigma_{x}p\left(x\right)lg\frac{p\left(x\right)}{q\left(x\right)},$$

In the resampling, the smaller particles are neglected and the larger particles are copied. The number of particles in the resampling is determined by KLD sampling in the process of particle duplication, and the number of particles of the next importance is determined, adjusting the number of particles on-line and reducing the computational complexity. The process of KLD sampling is shown in Algorithm 3.
**Algorithm 3.** KLD sampling algorithm**Input:**
$s_{t-1}=\{\langle x_{t-1}^{\left(i\right)},w_{t-1}^{\left(i\right)}\rangle |i=1,\ldots ,n\}$
, observations$\;z_{t}$
, limits ε 
and δ;**Output:**
$S_{t}$1: $S_{t}≔\emptyset ,n=0,k=0,\alpha =0$
   % initializing2: **do**                % generating samples3:  sampling from discrete distributions under the weight of known $s_{t-1}$
, the sequence is j(n)4:  sampling $x_{t}^{\left(n\right)}$
from$\;p(x_{t}|x_{t-1})$
using $x_{t-1}^{\left(j\left(n\right)\right)}$5:  $w_{t}^{\left(n\right)}≔p(z_{t}|x_{t}^{\left(n\right)})$       % calculate the importance weights6:  $\alpha \;≔\alpha +w_{t}^{\left(n\right)}$
         % update the normalization factor7:  $S_{t}≔S_{t}\cup^{\text{​}}\left\{\langle x_{t}^{\left(n\right)},w_{t}^{\left(n\right)}\rangle \right\}$    % insert the sample into the sample set8:  **if** ($x_{t}^{\left(n\right)}$ fall in bin b ) **then**    % update the number of bins9:   k≔k+110:   b≔non−empty11:  n≔n+1          % update the number of generated samples12: **while** ($n<\frac{1}{2\epsilon }X_{k-1,1-\delta }^{2}$)     % stop when come to the limits K-L with Equation (35)13: **for**
***i***: = 1, …, ***n***
**do**         % normalize importance weights14:  $w_{t}^{\left(i\right)}=w_{t}^{\left(i\right)}/\alpha$

## 4. Experimentation and Discussions

In order to validate the robustness and real-time performance of the two tracking algorithms (KF and adaptive PF), some experiments were carried out on the moving spherical target with or without occlusion, obstacles, different speeds and different trajectories. We tied the ball with a rope and pulled the string to move the ball.

### 4.1. Target Tracking with Occlusion and Obstacle in the Environment

**Figure 11:**
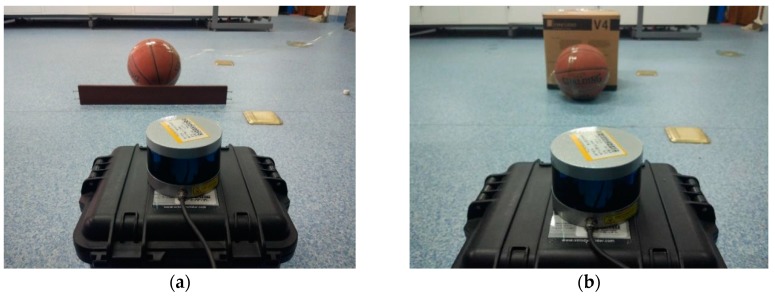
Experimental scenes: (**a**) Occlusion scene; (**b**) Obstacle scene.

**Figure 12:**
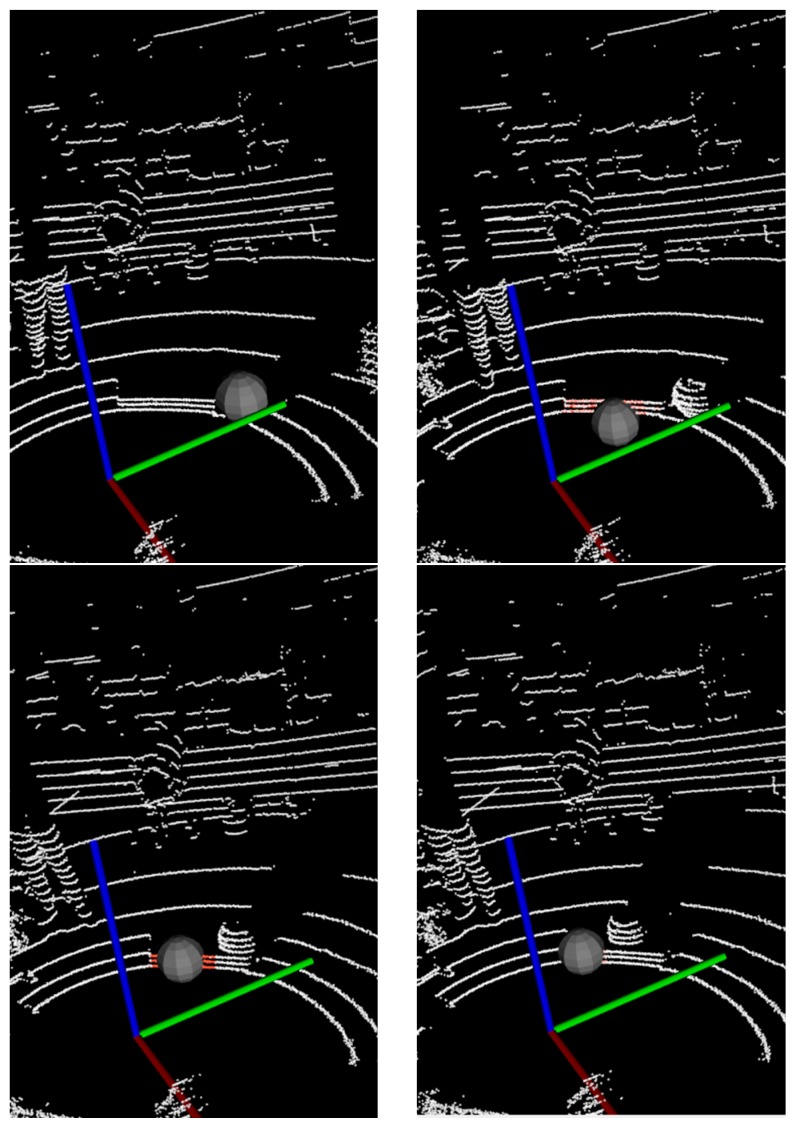
The tracking results with KF in occlusion scenes.

**Figure 13:**
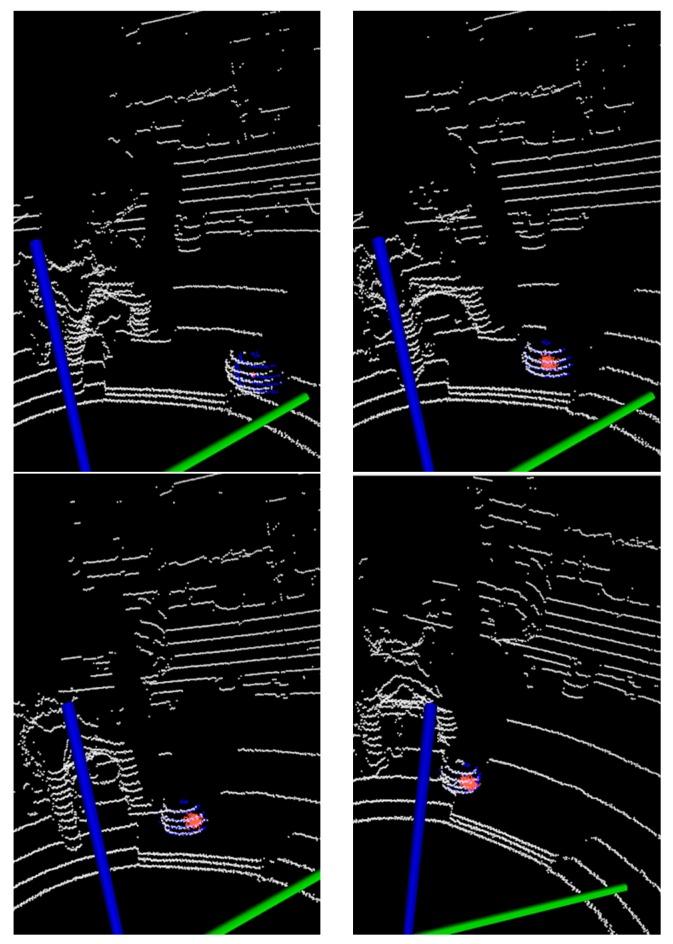
The tracking results with adaptive PF in occlusion scenes.

In target tracking processes, occlusion is a very common phenomenon. When the target is blocked, the valid information will be reduced and the tracking difficulty will be increased. Considering the partial occlusion of the spherical target with a long piece of wood, as shown in Figure 11a, the experimental results are shown in Figure 12 and Figure 13 with two different tracking algorithms, respectively.

In Figure 12, when the target is partially occluded, the KF clusters the wood point clouds together as the next frame’s measurement, and some tracking loss occurs. However, in Figure 13, the PF algorithm can track the object effectively where the occluded wood board does not cluster with the target.

**Figure 14:**
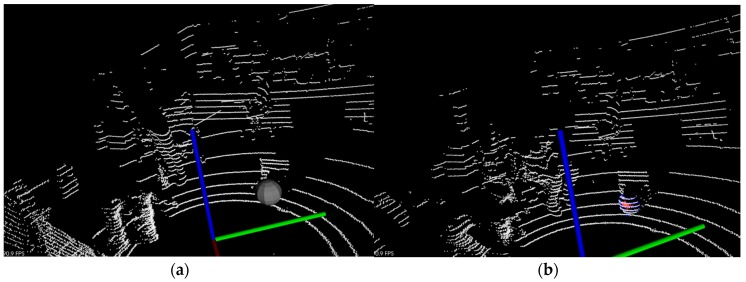
The tracking results in interference scenes: (**a**) Results with Kalman filter; (**b**) Results with adaptive particle filter.

Obstacles in front of the moving target are common in practical applications, and when multiple targets appear in motion, they may interfere with each other. The carton box as an obstacle is shown in Figure 11b. The experimental results are shown in Figure 14.

The two methods can track the spherical target continuously in the case of an obstacle. In Figure 14, the KF is susceptible to near point-cloud in the tracking process, and the PF has strong robustness for the motion tracking.

### 4.2. Target Tracking at Different Moving Speeds

**Figure 15:**
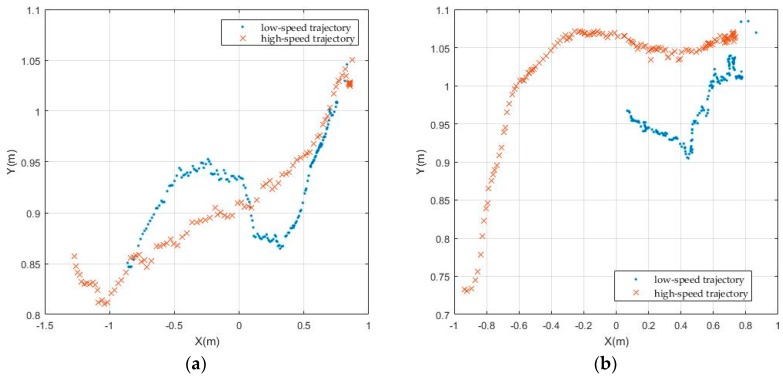
The tracking results at different moving speeds: (**a**) Results with KF; (**b**) Results with adaptive PF.

Whether the tracker can track moving targets at different speeds is also an important indicator of the performance. Due to the limitations of our laboratory environment and the point-cloud density of LiDAR equipment, the basketball position swings easily in the process of manually moving the basketball, especially at low speed, and a uniform speed can hardly be obtained. At the experimental process, the observed speed of movement of the basketball is 0.054 m/s at its low speed, and 0.125 m/s at its high speed. In Figure 15, the continuity of low-speed trajectory expressed by the light blue dots shows that the KF is more suitable for tracking at relative lower speeds. The continuity and density of high speed trajectory expressed by the red and brown fork shows the adaptive PF has better tracking performance. Basically, with more trials of different speed experiments, the adaptive PF has better tracking effects for the different moving speed than the KF.

### 4.3. Target Tracking in Different Motion Trajectories

**Figure 16:**
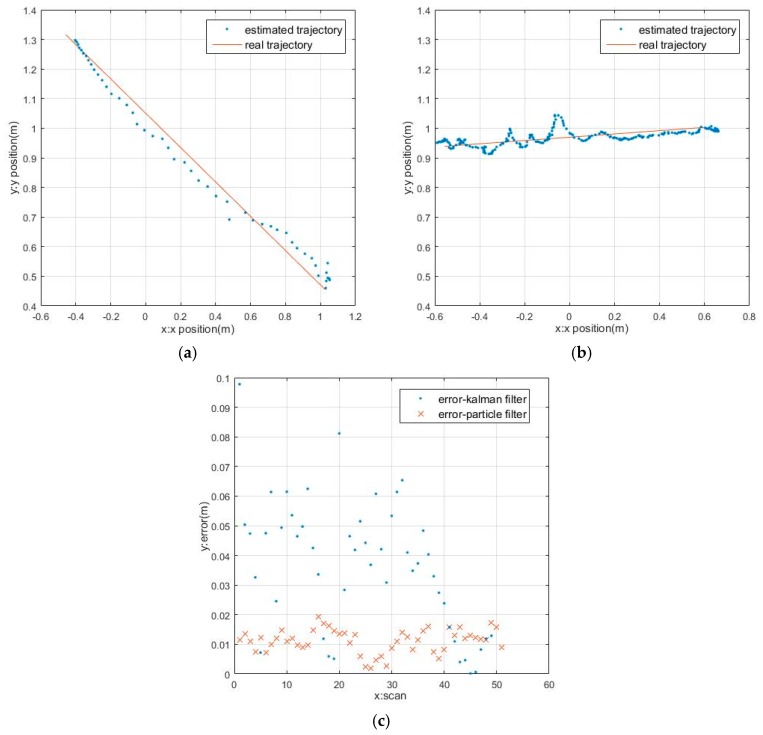
The tracking results in rectilinear motion: (**a**) Results with KF; (**b**) Results with adaptive PF; (**c**) The error of KF and PF.

**Figure 17:**
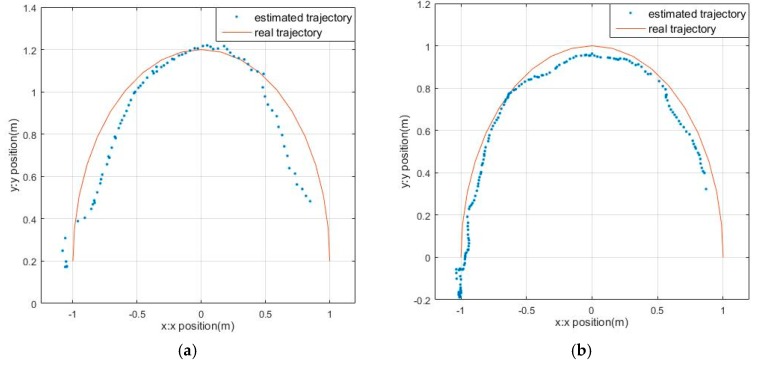

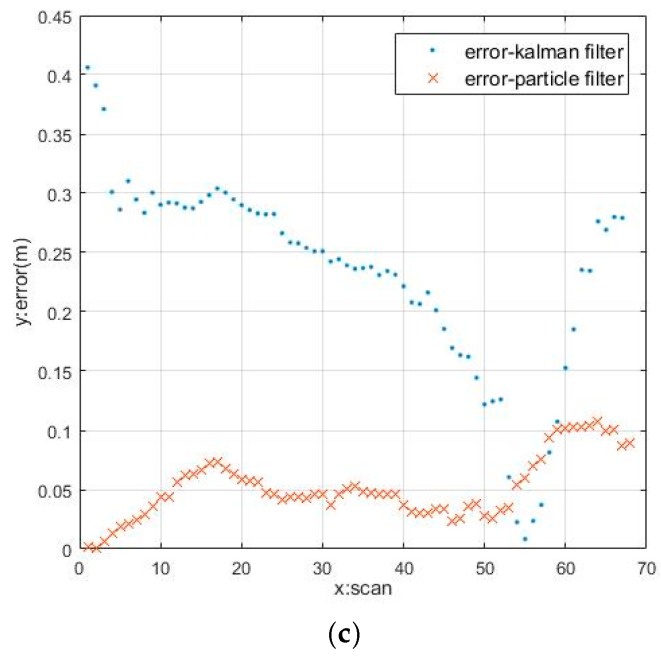
The tracking results in curvilinear motion: (**a**) Results with KF; (**b**) Results with adaptive PF; (**c**) The error of KF and PF.

In an actual situation, the target may move in a variety of trajectories. The spherical target’s real-time tracking is tested in three kinds of trajectories including straight line, curve and three-dimensional trajectory. The linear motion trajectory and error analysis are shown in Figure 16. Figure 16a,b show the actual trajectory and the estimated value of the spherical target in the rectilinear movement under the Kalman filter and the adaptive particle filter, respectively. The results indicate that the target moves as a curve in the same situation are shown in Figure 17. Obviously, the trajectory with the Kalman filter is smoother. However, the effect of PF tracking is better. The error comparison is shown in Figure 16c and Figure 17c. Errors and fluctuations of the KF are greater than the adaptive PF.

**Figure 18:**
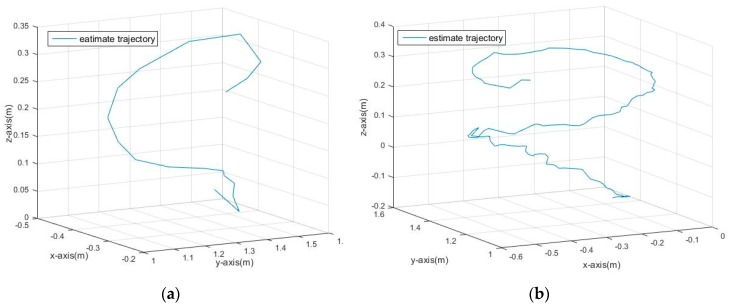
The tracking results in 3D space: (**a**) Results with KF; (**b**) Results with adaptive PF.

The results of the trajectory tracking of the spherical target in the three-dimensional space are shown in Figure 18. It is easy to see that the KF and the PF can basically track the moving target in three-dimensional space, but fluctuate in the *Z*-axis direction.

## 5. Conclusions

This paper has proposed a systematic real-time detection and tracking method for an indoor moving sphere using the VLP-16 3D LiDAR sensor. After a series of preprocessing steps of point-cloud data and global feature extraction, the Kalman filter and the adaptive particle filter method are used to estimate the real-time motion state of a spherical object. With three different kinds of indoor comparison experiments and analysis, the results show that the adaptive PF has better tracking performance. The specific work allows us to put forth two conclusions:

Firstly, the real-time detection of the spherical target is accomplished by acquiring the real-timepoint-cloud data of moving sphere at indoor, preprocessing with fast ground segmentation algorithm to remove outliers, and ground points clustering with Euclidean cluster algorithm, extracting target feature with VFH to establish model library and matching to detect spherical targets.

Secondly, the KF is used to real-time track the object, and the object position is estimated sequentially by real-time acquisition of the measured value, prediction and correction, while the adaptive PF is used to track the target, and the state of the target is estimated by sampling, calculating the weight and resampling. The efficiency of KF and adaptive PF in 3D lidar tracking is verified by indoor basketball tracking experiments, with a moving spherical object with or without occlusion and obstacles, respectively, at different speeds and over different trajectories. The experimental results show that adaptive PF has a small tracking error and strong robustness.

The motion tracking of a dynamic environment is one of the key components for intelligent agricultural harvesters to operate in real-world conditions. We will continue to exploit the 3D semantic perception with transfer learning and real-time location method of natural fruits for a better tracking performance.

## Figures and Tables

**Table 1:** Threshold Setting.

$T_{m}$	$T_{b}$	$T_{RMSE}$	$T_{dprev}$	$T_{ground}$
0.08	0.2	1	0.04	0.08
